# Case Series: Postoperative recurrent secondary hyperparathyroidism caused by ectopic submandibular parathyroid: 3 cases report and literature review

**DOI:** 10.3389/fendo.2026.1885648

**Published:** 2026-09-07

**Authors:** Tianyi Song, Jun Tian, Wentian Li, Jingxiu Li, Bingxun Pan, Zhonghui Li, Qingqing He, Peng Zhou

**Affiliations:** 1 Shandong First Medical University (Shandong Academy of Medical Sciences), Jinan, China; 2 Department of Thyroid & Breast Surgery, The 960th Hospital of the PLA Joint Logistics Support Force, Jinan, China; 3 Department of Nuclear Medicine, The 960th Hospital of the PLA Joint Logistics Support Force, Jinan, China; 4 Shandong University of Traditional Chinese Medicine, Jinan, China

**Keywords:** ^99m^Tc-MIBI SPECT/CT, case report, ectopic parathyroid gland, reoperation, secondary hyperparathyroidism, submandibular region

## Abstract

Postoperative recurrence for secondary hyperparathyroidism (SHPT) remains a clinical challenge. Ectopic parathyroid glands are a major cause of recurrence, and those located in the submandibular region are rare. This report retrospectively analyzes three patients on maintenance dialysis who developed recurrent elevation of parathyroid hormone (PTH) levels 3 to 37 months after parathyroidectomy (PTX) for SHPT. Neck ultrasound revealed no definite lesions; however, ^99m^Tc-methoxyisobutylisonitrile (^99m^Tc-MIBI) single-photon emission computed tomography/computed tomography (SPECT/CT) fusion imaging showed abnormal radioactive tracer uptake in the submandibular region, suggestive of ectopic parathyroid glands. All three patients underwent a second or multiple surgeries, with complete excision of the submandibular masses. Postoperative pathology confirmed parathyroid tissue. After surgery, PTH levels returned to the normal range, and clinical symptoms improved markedly. Combined with a literature review, this article discusses the causes of recurrence after SHPT surgery, the anatomical basis of ectopic lesions in the submandibular region, and the localization diagnosis and treatment strategies for ectopic parathyroid glands, aiming to improve clinical awareness of such rare cases.

## 1 Introduction

Secondary hyperparathyroidism (SHPT) is a common complication of chronic kidney disease (CKD) (1). It is characterized by abnormal parathyroid tissue proliferation and elevated parathyroid hormone (PTH) levels, often accompanied by persistent hyperphosphatemia, hypercalcemia, or hypocalcemia. These abnormalities can lead to multisystem involvement, including skin, bone, and cardiovascular diseases, severely affecting patients’ quality of life and long-term survival. For patients with refractory or progressive SHPT who fail medical therapy, parathyroidectomy (PTX) remains the most effective treatment (2). However, postoperative recurrence rates remain high, ranging from 10% to 30% (3). Preoperative missed diagnosis and incomplete intraoperative resection of ectopic parathyroid glands are the primary causes of recurrence (4). Postoperative recurrence is generally defined as elevated PTH levels after an initial period of normalization following surgery, whereas persistent disease refers to failure of PTH to normalize within 6 months postoperatively (1). In this report, all three cases presented with recurrent disease, as evidenced by an initial postoperative decrease in PTH followed by subsequent elevation.

A large meta-analysis including more than 7,000 individuals from healthy populations and patients with hyperparathyroidism reported a prevalence of ectopic parathyroid glands of approximately 15.9% (5). Common ectopic locations include the thymus, intrathyroidal sites, and the carotid sheath (6). However, ectopic parathyroid glands located in the submandibular region are rare, and most reports in the literature are case reports (7). Due to their unusual location, these lesions are easily missed on conventional imaging, leading to repeated surgeries and increased clinical difficulty. This study retrospectively analyzes three patients with recurrent SHPT after PTX, in whom ectopic parathyroid glands in the submandibular region were confirmed by ^99m^Technetium-methoxyisobutylisonitrile single-photon emission computed tomography/computed tomography (^99m^Tc-MIBI SPECT/CT). The study also reviews the literature to discuss the clinical characteristics, localization diagnosis, and surgical management of such cases, aiming to improve clinical awareness of this rare condition.

## 2 Literature review methodology

A systematic literature search was performed in PubMed, Web of Science, and CNKI (China National Knowledge Infrastructure) databases from inception to August 2025. The search strategy combined the following keywords: “secondary hyperparathyroidism” OR “renal hyperparathyroidism” AND “ectopic parathyroid” OR “submandibular parathyroid” OR “undescended parathyroid” AND “reoperation” OR “recurrence.” Inclusion criteria were: (a) case reports or case series reporting ectopic parathyroid glands in the submandibular region in patients with SHPT; (b) confirmation by histopathology; and (c) availability of clinical and imaging data. Exclusion criteria were: (a) primary or tertiary hyperparathyroidism; (b) ectopic glands located outside the submandibular region; and (c) non-English or non-Chinese language publications without available translation. Reference lists of included articles were also manually screened for additional relevant publications.”

The initial search yielded 47 articles. After removing duplicates (n=12), 35 articles underwent title and abstract screening, of which 19 were excluded as irrelevant. The remaining 16 articles were assessed in full text, and 6 case reports met the inclusion criteria. All identified cases are cited and discussed in the relevant sections of this manuscript. Given the limited number of published cases, the key characteristics of previously reported submandibular ectopic parathyroid glands are summarized in the Discussion rather than presented as a separate table.

## 3 Case presentation

**Figure 1:**
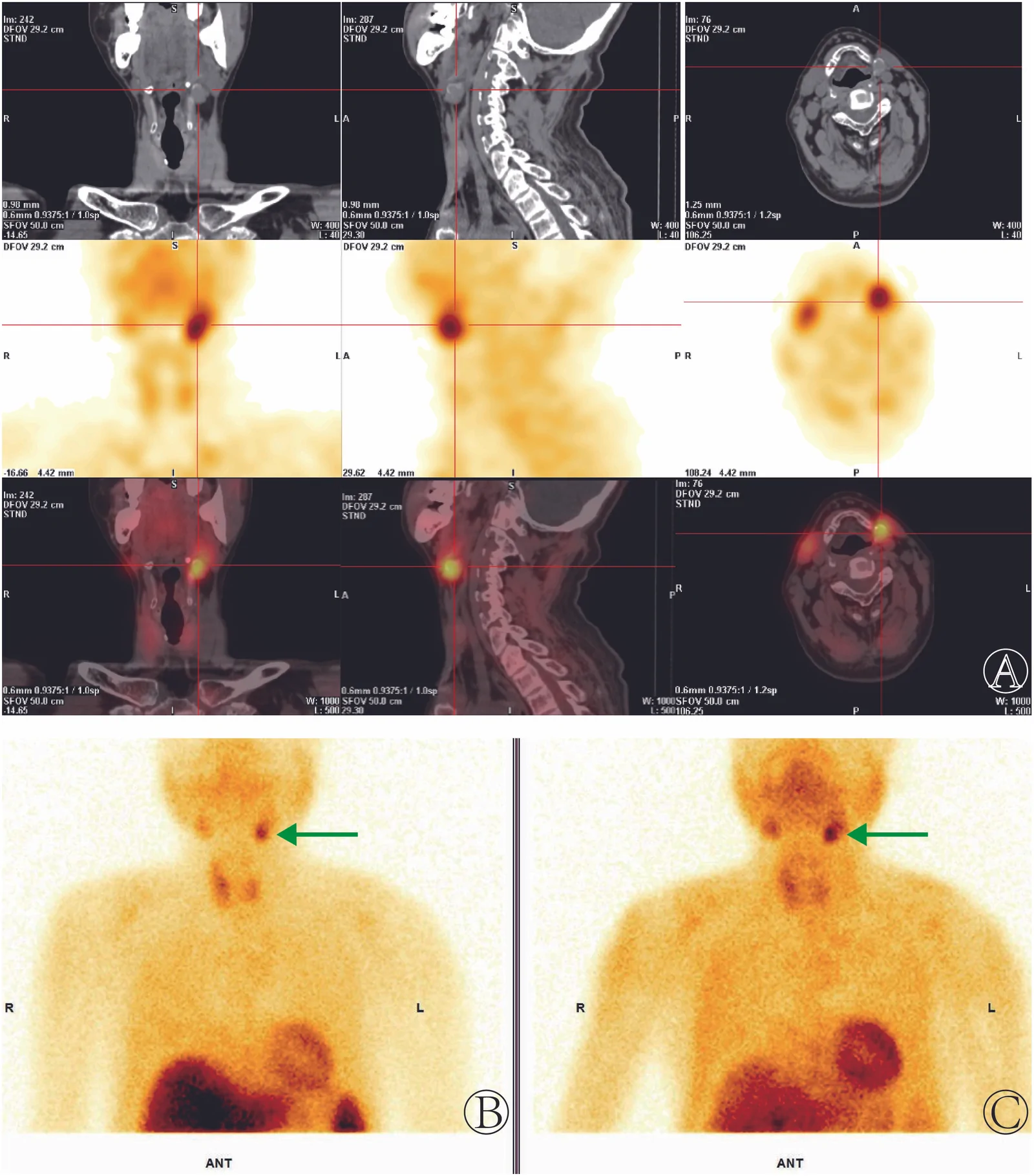
^99m^Tc-MIBI SPECT/CT fusion images and early and late planar images of ^99m^Tc-MIBI SPECT for case 1. **(A)** First row: Three-dimensional CT scan images; second row: ^99m^Tc-MIBI SPECT images; third row: ^99m^Tc-MIBI SPECT/CT fusion images. The first, second, and third columns show the coronal, sagittal, and transverse planes, respectively. **(B)** Early (20 min) planar image of ^99m^Tc-MIBI SPECT: Increased tracer uptake (arrow) in a nodule in the medial left submandibular region (level IIa). The thyroid gland is visible inferiorly. **(C)** Late (2 h) planar image of ^99m^Tc-MIBI SPECT: Persistent increased tracer uptake (arrow) in the nodule in the medial left submandibular region (level IIa). Tracer uptake in the thyroid gland is generally decreased compared with the early image.

**Figure 2:**
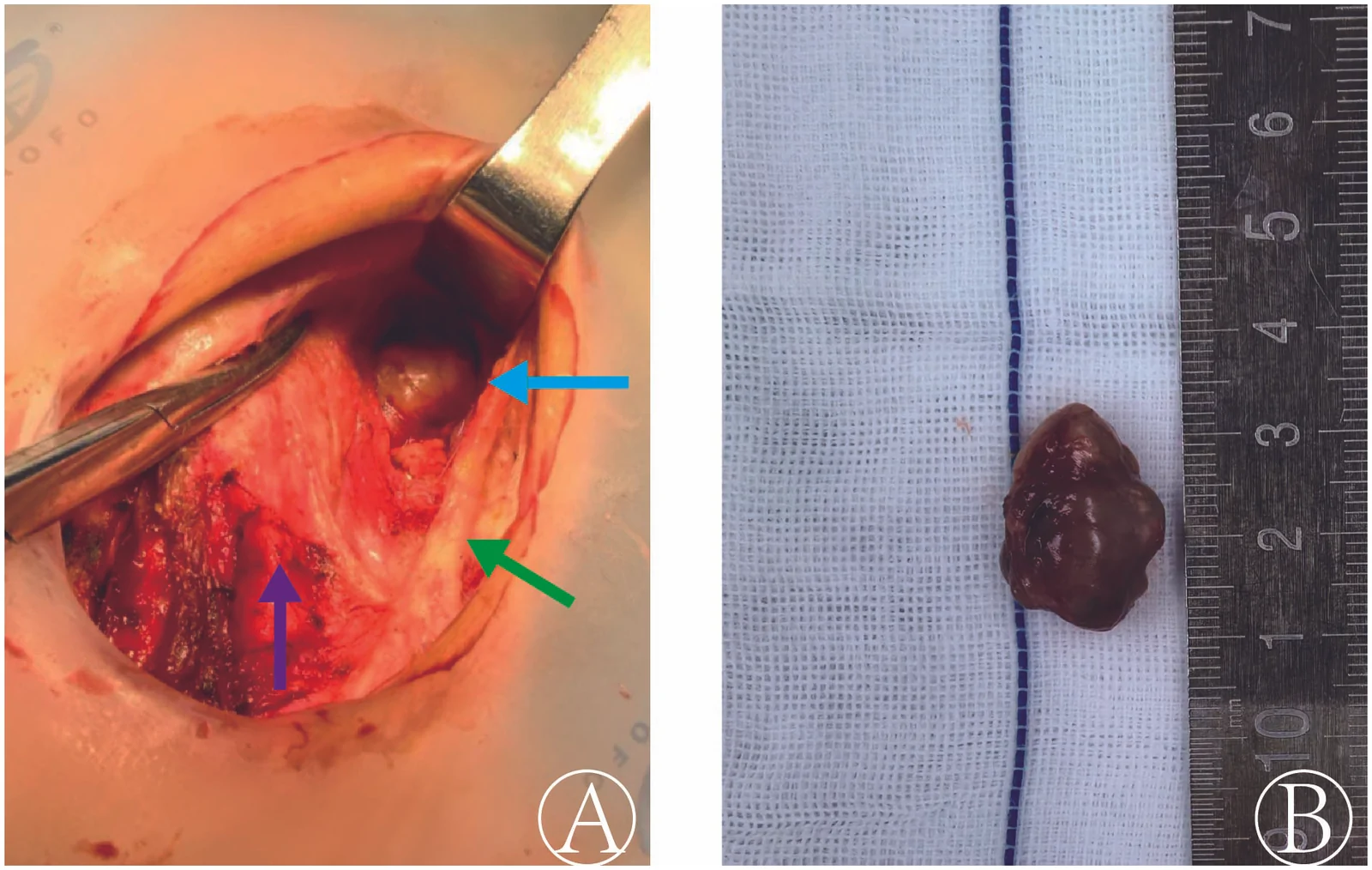
Intraoperative findings and the resected ectopic parathyroid gland from case 1. **(A)** Intraoperative view through an anterior cervical incision. The blue arrow indicates the ectopic parathyroid gland; the purple arrow points to the anterior aspect of the trachea, indicating the thyroid cartilage; the green arrow indicates the left sternocleidomastoid muscle. **(B)** Gross specimen of the resected ectopic parathyroid gland.

The clinical characteristics of three patients are presented in **Table 1**. Case 1 was selected as a representative case to illustrate the imaging findings and surgical procedures (**Figures 1**, **2**).

**Table 1:** Clinical characteristics of three patients with ectopic parathyroid glands in the submandibular region.

Characteristics	Case 1	Case 2	Case 3
Gender	Male	Male	Male
Age (years)	53	34	34
Duration of dialysis (years)	14	5	9
Previous surgical procedures	Total PTX + left forearm AT; Left forearm graft excision	Total PTX	Total PTX
Time to recurrence after first surgery (months)	3	18	37
Ultrasound findings	Negative for lesion	Hypoechoic mass in right level I	Negative for lesion
Preoperative Ca (mmol/L)	2.16	2.31	2.56
Preoperative P (mmol/L)	1.89	1.97	2.56
Preoperative ALP (U/L)	245	312	189
Preoperative PTH (pg/mL)	3187.0	2251.0	1847.0
Intraoperative PTH (pg/mL)	215.0	273.0	439.0
^99m^Tc-MIBI SPECT/CT localization	Medial left submandibular region	Medial right submandibular region	Medial left submandibular region
Specimen size (cm)	2.2*1.6*1.4	3.8*3.0*1.0	1.8*1.5*0.6
Pathology	Parathyroid nodular hyperplasia	Parathyroid nodular hyperplasia	Parathyroid nodular hyperplasia
Postoperative day 3 Ca (mmol/L)	1.86	1.67	2.02
Postoperative day 3 PTH (pg/mL)	6.0	17.2	68.3
Postoperative 3-month PTH (pg/mL)	21.5	28.3	45.8
Symptom improvement	Marked	Marked	Marked

Duration of dialysis refers to the duration of preoperative dialysis at the time of reoperation; Preoperative, intraoperative, and postoperative day 3 PTH are those measured during the reoperation; Specimen size was measured from the gross specimen.Abbreviations: ALP, alkaline phosphatase. PTH, Parathyroid hormone; ^99m^Tc-MIBI, ^99m^Technetium-methoxyisobutylisonitrile; PTX, Parathyroidectomy; AT, Autotransplantation.

### 3.1 Case 1

A 53-year-old man with a 14-year history of end stage renal disease (ESKD) on regular peritoneal dialysis underwent total PTX with left forearm autotransplantation (AT) at another hospital in October 2021 for SHPT (PTH 1369.4 pg/mL) presenting with bone pain and pruritus. The surgical approach was a standard open anterior cervical approach. The intraoperative PTH level was 328.5 pg/mL, representing a 76% decrease. Four glands were reportedly identified and resected: the right upper, right lower, and left lower were submitted for pathology, while the left upper was autotransplanted. Postoperative pathology of the resected parathyroid glands (right lower, right upper, and left lower) showed parathyroid nodular hyperplasia. On postoperative day 3, PTH decreased to 139.8 pg/mL, and his symptoms resolved.

In January 2022, follow-up revealed a progressive increase in PTH to 1405.9 pg/mL, and the patient underwent left forearm graft excision at the same hospital. The intraoperative PTH level was 1052.0 pg/mL, and pathology of the excised specimen showed a small amount of parathyroid tissue. Over the following three years, his PTH remained persistently above 2000 pg/mL despite treatment with calcitriol and cinacalcet. Bone pain and pruritus recurred, and PTH was 2463.8 pg/mL.

In August 2025, the patient was admitted to our hospital with a diagnosis of recurrent elevation of PTH more than one year after PTX. Neck ultrasound revealed no definite parathyroid lesion. Laboratory tests showed PTH 3187.0 pg/mL and calcium (Ca) 2.16 mmol/L. ^99m^Tc-MIBI SPECT/CT (**Figure 1**) demonstrated increased tracer uptake in a nodule in the medial left submandibular region (level IIa), suggestive of parathyroid hyperplasia. After multidisciplinary discussion, the patient underwent excision of the left ectopic parathyroid gland under general anesthesia. Intraoperatively, a suspected parathyroid gland was identified medial to the left submandibular gland (intraoperative findings and gross specimen shown in **Figure 2**). The intraoperative PTH level dropped to 215.0 pg/mL. Postoperative pathology confirmed parathyroid nodular hyperplasia.

After surgery, the patient’s bone pain and pruritus improved markedly. On postoperative day 3, PTH was 6.0 pg/mL, Ca 1.86 mmol/L, and phosphorus (P) 0.63 mmol/L. At the 3-month follow-up, PTH was 21.5 pg/mL, Ca 1.58 mmol/L, and P 0.76 mmol/L.

### 3.2 Case 2

A 34-year-old man with a 5-year history of ESKD on regular hemodialysis underwent total PTX without AT at our hospital in June 2024 for SHPT. Preoperatively, he had been on hemodialysis for 4 years, and elevated PTH (1590.0 pg/mL) had been noted for more than 8 months. Preoperative ^99m^Tc-MIBI SPECT/CT showed increased tracer uptake in nodules located posterior to the upper pole and inferolateral to the lower pole of the left lobe, as well as posterior to the body and lower pole of the right lobe, suggestive of parathyroid hyperplasia. Contrast-enhanced neck CT was not performed before the initial operation because the institutional preoperative imaging protocol at that time primarily consisted of neck ultrasonography and ^99m^Tc-MIBI SPECT/CT. As the SPECT/CT examination demonstrated multiple presumed hyperplastic glands in their usual anatomical locations and did not indicate an ectopic submandibular lesion, no additional cross-sectional imaging was obtained. A standard open anterior cervical approach was performed. The intraoperative PTH level was 1072.0 pg/mL. Three glands were identified and resected (left upper, left lower, and right lower). Postoperative pathology of the resected glands confirmed parathyroid nodular hyperplasia. On postoperative day 3, PTH was 1080.0 pg/mL, and his symptoms did not improve.

In December 2025, the patient noticed an abnormal mass in the neck and returned to our hospital for further evaluation. He was admitted with a diagnosis of an abnormal neck mass detected on physical examination after PTX for SHPT. Neck ultrasound revealed a hypoechoic mass (30*18 mm) in the right level I region near the hyoid bone, suspicious for an enlarged lymph node. Laboratory tests showed PTH 2251.0 pg/mL and Ca 2.31 mmol/L. Fine-needle aspiration cytology of the mass revealed many cells with scant cytoplasm, oval shape, and high nuclear-to-cytoplasmic ratio, suggesting parathyroid origin. The washout fluid showed PTH >5000 pg/mL. ^99m^Tc-MIBI SPECT/CT demonstrated increased tracer uptake in a nodule in the medial right submandibular region (level IIa), suggestive of parathyroid hyperplasia.

The patient underwent excision of the right cervical mass under general anesthesia. Intraoperatively, a suspected parathyroid gland was identified medial to the right submandibular gland. The intraoperative PTH level dropped to 273.0 pg/mL. Postoperative pathology of the ectopic parathyroid gland confirmed parathyroid nodular hyperplasia.

After surgery, the patient’s symptoms improved markedly. On postoperative day 3, PTH was 17.2 pg/mL, Ca 1.67 mmol/L, and P 1.50 mmol/L. At the 3-month follow-up, PTH was 28.3 pg/mL, Ca 1.48 mmol/L, and P 1.59 mmol/L.

### 3.3 Case 3

A 34-year-old man with a 9-year history of ESKD on regular hemodialysis underwent total PTX without AT at our hospital in December 2021 for secondary SHPT. Preoperatively, he had been on hemodialysis for 5 years, and elevated PTH (3458.0 pg/mL) had been noted for more than 1 year. Preoperative ^99m^Tc-MIBI SPECT/CT showed mildly increased focal tracer uptake in the parathyroid regions at the upper and lower poles of both thyroid lobes, suggestive of parathyroid hyperplasia or adenoma. Contrast-enhanced neck CT was not performed before the initial operation for the same reason: the initial ^99m^Tc-MIBI SPECT/CT demonstrated multiple presumed hyperplastic glands in their usual anatomical locations, without a suspicious focus in the submandibular region. A standard open anterior cervical approach was performed. The intraoperative PTH level was 439.0 pg/mL. Four glands were identified and resected (left upper, left lower, right upper, and right lower). Postoperative pathology of the resected glands confirmed parathyroid nodular hyperplasia. On postoperative day 3, PTH was 291.8 pg/mL, and his symptoms improved.

In December 2025, a routine physical examination revealed elevated PTH. The patient returned to our hospital for further evaluation and was admitted with a diagnosis of recurrent elevation of PTH more than one year after PTX for SHPT. Neck ultrasound showed no definite parathyroid lesion. Laboratory tests revealed PTH 1847.0 pg/mL and Ca 2.56 mmol/L. ^99m^Tc-MIBI SPECT/CT demonstrated increased tracer uptake in a nodule in the medial left submandibular region (level IIa), suggestive of parathyroid hyperplasia.

The patient underwent excision of the left ectopic parathyroid gland under general anesthesia. Intraoperatively, a suspected parathyroid gland was identified medial to the left submandibular gland. The intraoperative PTH level dropped to 354.0 pg/mL. Postoperative pathology of the ectopic parathyroid gland (left lateral cervical region) confirmed parathyroid nodular hyperplasia.

After surgery, the patient’s symptoms improved markedly. On postoperative day 3, PTH was 68.3 pg/mL, Ca 2.02 mmol/L, and P 2.58 mmol/L. At the 3-month follow-up, PTH was 45.8 pg/mL, Ca 1.60 mmol/L, and P 2.57 mmol/L.

## 4 Discussion

### 4.1 Analysis of causes of recurrence after PTX

Recurrence after PTX for SHPT is an important clinical issue, with reported rates ranging from 10% to 30%, which significantly affects the long-term prognosis of patients with ESKD (3). The causes of recurrence can be summarized into three main categories: First, incomplete surgical resection, leaving functional parathyroid tissue in the neck; Second, excessive hyperplasia of autotransplanted parathyroid tissue under the persistent uremic environment; Third, the presence of ectopic or supernumerary parathyroid glands that were not detected by preoperative imaging or clinical evaluation. These residual or ectopic glands may undergo hyperplasia driven by persistent calcium-phosphate metabolism disorders in ESKD, becoming a source of abnormal PTH secretion (8).

Among the three patients reported here, Case 1 had undergone a second surgery for forearm graft hyperplasia, yet his PTH level remained persistently above 2000 pg/mL, far exceeding the upper limit typically associated with graft hyperplasia (usually below 1000 pg/mL) (9). In our three patients, preoperative PTH levels before the second or repeat surgery were 3187.0, 2251.0, and 1847.0 pg/mL, respectively, all significantly above this range, suggesting that graft hyperplasia alone could not explain the markedly elevated PTH levels. Furthermore, all three patients had undergone total PTX during their first surgery, and postoperative neck ultrasound revealed no definite residual tissue. Taken together, these findings essentially rule out residual neck tissue or forearm graft hyperplasia as the primary cause of recurrence, and support that the ectopic parathyroid glands in the submandibular region were the underlying cause of recurrence in these cases.

### 4.2 Embryological basis of ectopic parathyroid glands

Ectopic parathyroid glands result from anatomical variations occurring during the embryonic migration of the parathyroid glands (10). The parathyroid glands originate from the third and fourth pharyngeal pouches and descend to their normal anatomical positions at the dorsal aspects of the upper and lower poles of the thyroid gland around the sixth week of embryonic development. The inferior parathyroid glands, which derive from the third pharyngeal pouch, migrate a longer distance downward together with the thymus. This longer migration pathway makes them more prone to ectopia compared with the superior parathyroid glands, which originate from the fourth pharyngeal pouch. Clinically, ectopic parathyroid glands are most commonly found in the thymus (accounting for approximately 40% of ectopic cases), followed by intrathyroidal locations and the carotid sheath (6).

Ectopic parathyroid glands in the submandibular region, however, are rare. This location corresponds to the starting point of parathyroid embryonic migration; complete arrest of migration at this stage results in a submandibular ectopic parathyroid gland, which is the core embryological reason for the rarity of such cases. The submandibular region has a complex anatomy. Ectopic parathyroid glands may be located within the submandibular gland parenchyma, superficial or deep to the submandibular gland, or between the anterior and posterior bellies of the digastric muscle (11). In our three cases, all ectopic glands were located in the medial submandibular region (level IIa). This area is a concealed region within the cervical lymph node classification and is partially obscured by the submandibular gland. On conventional neck ultrasound, these lesions can be easily missed due to acoustic shadowing or may be mistaken for enlarged lymph nodes. For example, in Case 2, the initial ultrasound diagnosis was a “hypoechoic mass in the right level I region near the hyoid bone, suspicious for an enlarged lymph node.” This anatomical feature is an important factor contributing to the preoperative missed diagnosis of ectopic lesions in this region.

Exposure of the medial submandibular region (level IIa) is challenging, and this area is closely adjacent to important structures, including the lingual nerve, hypoglossal nerve, submandibular duct, and facial artery (12). Surgical manipulation in this region carries a risk of injury to these structures due to retraction or dissection. In all three of our cases, a “capsule-focused fine dissection” technique was used, and no major structural injuries occurred. This suggests that surgery in this region should center on the lesion with gradual dissection along the capsule, avoiding blind extension of the surgical field. Preoperative clarification of the anatomical relationship between the lesion and surrounding structures is essential to provide precise guidance for intraoperative dissection.

### 4.3 Localization diagnosis and treatment of ectopic parathyroid glands

For patients with recurrent SHPT after PTX, accurate preoperative imaging localization is critical for successful reoperation. Conventional neck ultrasound and CT have a high detection rate for hyperplastic parathyroid glands in common locations, but they often fail to detect ectopic glands, particularly those in unusual sites such as the submandibular region (13). ^99m^Tc-MIBI SPECT/CT fusion imaging is currently the preferred imaging modality for diagnosing ectopic hyperparathyroidism. In the three cases reported here, neck ultrasound detected no definite lesion in two cases and misidentified the lesion as an enlarged lymph node in one case, whereas ^99m^Tc-MIBI SPECT/CT accurately localized all ectopic glands to the medial submandibular region (level IIa). For patients with recurrent SHPT and negative ultrasound or CT findings, ^99m^Tc-MIBI SPECT/CT is particularly valuable because it provides both functional and anatomical information, helping to distinguish physiological uptake in the submandibular gland from pathological tracer accumulation in ectopic lesions. This improves preoperative localization accuracy, enables precise intraoperative exploration and resection of all lesions, shortens operative time, reduces the difficulty of reoperation, and increases the success rate of surgery (14). In retrospect, the initial scintigraphic findings in Cases 2 and 3 also illustrate an important limitation of ^99m^Tc-MIBI imaging in multiglandular SHPT. Radiotracer uptake may vary among hyperplastic glands according to gland size, cellular composition, mitochondrial content, and the degree of hyperplasia. More intense uptake by larger glands may reduce the relative conspicuity of a smaller or less radiotracer-avid ectopic gland in an unusual location. Following removal of the dominant glands, persistent stimulation in the uremic environment may have promoted further enlargement and functional activation of the residual ectopic tissue. These factors probably increased the relative conspicuity of the submandibular lesions on subsequent SPECT/CT examinations. However, because the initial ectopic glands were not histologically assessed and the serial scintigraphic changes were evaluated retrospectively, this explanation remains presumptive. In the context of SHPT, thyroid abnormalities, including nodular goiter, thyroiditis, and thyroid dysfunction, are common in patients with chronic kidney disease and may confound the interpretation of parathyroid imaging. For example, thyroid nodules may be mistaken for parathyroid lesions on ultrasound, and thyroid pathology may affect tracer uptake on ^99m^Tc-MIBI scintigraphy. In patients with recurrent or persistent SHPT, a thorough evaluation of thyroid function and morphology should be considered as part of the preoperative assessment, particularly when imaging findings are inconclusive. This integrated approach aligns with the multidisciplinary management paradigm emphasized by Guo (15) and ensures that coexisting endocrine pathologies are not overlooked. In recent years, emerging imaging modalities have shown promising utility in ectopic parathyroid localization. ¹^8^F-fluorocholine PET/CT has demonstrated superior sensitivity for parathyroid adenoma detection, particularly in patients with negative conventional imaging (13). Four-dimensional CT (4D-CT), which adds a temporal component to characterize perfusion patterns of parathyroid tissue, has also shown high sensitivity for ectopic gland localization. However, these advanced modalities may not be widely available in all centers, and their cost-effectiveness in routine SHPT evaluation requires further investigation.

For well-localized ectopic parathyroid glands in the submandibular region, surgery remains the only effective treatment to correct calcium-phosphate metabolism disorders and control abnormal PTH levels (8). Precise preoperative localization using ^99m^Tc-MIBI SPECT/CT and meticulous intraoperative dissection are essential for surgical success. Due to the unique location of the submandibular region, which is adjacent to important nerves, blood vessels, and glandular ducts, delicate surgical techniques are required. In all three cases, intraoperative parathyroid hormone assay (IOPTH) was used during reoperation, with PTH levels measured 20 minutes after excision of the parathyroid gland. The results showed that PTH levels dropped by more than 80% in Cases 1 and 2, and by nearly 80% in Case 3, indicating complete resection of the hyperfunctioning lesions. IOPTH effectively guided the termination of surgery and avoided unnecessary extensive exploration (16). Postoperatively, PTH levels normalized in all patients, and symptoms such as bone pain and pruritus were markedly relieved. All three patients in this series developed varying degrees of hungry bone syndrome (HBS) postoperatively, presenting with hypocalcemia (POD3 Ca: 1.86, 1.67, and 2.02 mmol/L, respectively). HBS is a well-recognized complication following PTX in patients with severe SHPT, characterized by profound and prolonged hypocalcemia due to rapid skeletal calcium uptake after suppression of high bone turnover (17). In our patients, management of HBS included: (a) Intravenous calcium supplementation: continuous infusion of calcium gluconate (1–2 g/day) was initiated when serum calcium dropped below 1.9 mmol/L, with dose titration based on frequent (every 4–6 hours) calcium monitoring; (b) Oral calcium and active vitamin D: high-dose oral calcium carbonate (up to 3–6 g/day) and calcitriol (0.5-1.0 μg/day) were administered once oral intake was tolerated; and (c) Phosphate management: phosphate binders were reduced or temporarily withheld to prevent exacerbation of hypocalcemia. However, all three patients continued to require oral calcium and calcitriol supplementation at the 3-month follow-up, underscoring the prolonged nature of HBS in dialysis-dependent patients. These observations highlight the importance of a structured, multidisciplinary approach to postoperative calcium management, including close biochemical monitoring and patient education regarding adherence to supplementation.

## 5 Conclusion

In summary, for patients with recurrent SHPT after PTX, the possibility of an ectopic parathyroid gland should be considered (18). When conventional ultrasound and CT findings are negative, ^99m^Tc-MIBI SPECT/CT imaging should be actively pursued. Although the submandibular region is a rare location for ectopic parathyroid glands, it should not be overlooked. Once an ectopic lesion is identified, surgical excision is key to correcting biochemical abnormalities, relieving clinical symptoms, and improving patients’ quality of life. For patients undergoing initial PTX for SHPT, when performing ^99m^Tc-MIBI SPECT/CT, careful scrutiny should extend beyond the routine examination of the dorsal aspects of the upper and lower thyroid poles to include detailed exploration of rare ectopic sites such as the submandibular region and carotid sheath. This approach may help reduce postoperative recurrence rates and avoid second or multiple surgeries. By reporting three cases of ectopic parathyroid glands in the rare submandibular region, we enrich the clinical understanding of the causes of recurrence after SHPT surgery and provide a reference for the diagnosis and treatment of similar cases. This study is a small case series with a relatively short follow-up period. Although short-term outcomes are favorable, the long-term recurrence rate after excision of submandibular ectopic glands remains unknown. Extended follow-up is ongoing to further evaluate the durability of the surgical response and to monitor for potential recurrence from other ectopic sites.

## Data Availability

The raw data supporting the conclusions of this article will be made available by the authors, without undue reservation.
